# Dysarthria and idiopathic Parkinson's disease: a current overview of the relationship between speech impairment and quality of life

**DOI:** 10.1055/s-0046-1829126

**Published:** 2026-10-23

**Authors:** Ana Luiza Fabris Moraes, Vivian Kaori Segawa, Arthur Jun Deguti Hata, Alice Estevo Dias, Margarete de Jesus Carvalho

**Affiliations:** 1 Centro Universitário Faculdade de Medicina do ABCSanto André SPBrazil; 2 Universidade de São Paulo, Faculdade de Medicina, Departamento de NeurologiaSão Paulo SPBrazil; 3 Centro Universitário Faculdade de Medicina do ABC, Ambulatório de Distúrbios do MovimentoSanto André SPBrazil

**Keywords:** Parkinson Disease, Dysarthria, Quality of Life

## Abstract

Idiopathic Parkinson's disease (IPD) is a chronic and progressive neurodegenerative disorder characterized by resting tremor, rigidity, bradykinesia, and impairment of automatic movements. Dysarthria is one of the most common manifestations, significantly impacting communication and quality of life (QoL). The Living with Dysarthria (LwD) questionnaire is a tool developed to assess the functional and psychosocial impact of this condition, providing a patient-centered measure of symptom severity. This article aims to present a literature review on the impact of dysarthria in IPD and the use of the LwD questionnaire in evaluating patients' QoL. Five studies investigating dysarthria in IPD and its impact on QoL using the LwD were selected, with searches conducted in the SciELO and PubMed databases, including one study presented at a conference in 2024. The analyzed studies showed that dysarthria compromises not only speech but also emotional and social aspects, directly affecting self-esteem and social participation. The evidence reinforces the importance of integrated therapeutic approaches that address both motor and psychosocial limitations of communication. Furthermore, there is a need for new studies using the LwD specifically in IPD patients to strengthen the scientific basis and improve intervention and therapeutic strategies.

## INTRODUCTION

Idiopathic Parkinson's disease (IPD) is the second most prevalent neurodegenerative disorder worldwide, surpassed only by Alzheimer's disease. 1

This disease is characterized by the progressive degeneration of dopaminergic neurons in the substantia nigra, leading to a range of motor symptoms, including tremor, bradykinesia, rigidity, and gait disturbances. Although traditionally defined as a movement disorder, it is also associated with a broad spectrum of non-motor manifestations, such as intestinal constipation, urinary dysfunction, arterial hypotension, cognitive impairment, depression, and sleep disturbances. In addition to these features, patients frequently develop speech impairments, particularly dysarthria, which represents a significant yet often underrecognized clinical manifestation of the disease. 2 3 4 5

Furthermore, IPD is part of the group of synucleinopathies, a set of neurodegenerative diseases characterized by the abnormal deposition of the α-synuclein protein in neurons, nerve fibers, and glial cells. This aggregation leads to the formation of Lewy bodies, which are considered the primary pathological hallmark of the group. The accumulation of Lewy bodies precedes signs of neuronal degeneration, reflecting a progressive neurodegenerative process that extends across different regions of the nervous system. Among these, the substantia nigra's pars compacta stands out, whose degeneration results in the loss of dopaminergic neurons and the manifestation of typical symptoms of the disease, with severity tending to correlate with the extent of the lesion. 6 7 8

Dysarthria is a neurological speech disorder that compromises articulation, phonation, and speech intelligibility, ultimately affecting communication effectiveness. Given that speech is a fundamental component of social interaction and personal expression, these impairments can have a substantial negative impact on patients' quality of life (QoL). 9

In this context, the use of patient-reported outcome measures has gained increasing relevance, as they allow for a more comprehensive assessment of the patient's perspective. The Living with Dysarthria (LwD) questionnaire is a specific instrument designed to evaluate the psychosocial impact of dysarthria, enabling the identification of patient-perceived communication difficulties, guiding individualized speech-language interventions, and supporting the monitoring of therapeutic outcomes. Furthermore, its use contributes to a more patient-centered approach, addressing not only motor symptoms but also broader aspects of QoL.

Despite its potential clinical utility, the application of the LwD questionnaire in IPD remains insufficiently explored in the literature. Therefore, this study aims to review the existing evidence on the use of the LwD questionnaire in individuals with IPD and to discuss its potential clinical implications.

### Dysarthria in IPD

According to the Movement Disorder Society's (MDS) 2015 Clinical Diagnostic Criteria, IPD is defined by the presence of parkinsonism, characterized by bradykinesia in combination with resting tremor or rigidity. Although speech impairment is not a mandatory diagnostic criterion, it represents a frequent and clinically relevant manifestation of the disease, with significant impact on patient functioning and QoL.

In this context, dysarthria emerges as a common motor manifestation of IPD, reflecting impairments in the neuromuscular control of speech production. Hypokinetic dysarthria, the most prevalent subtype associated with IPD, is characterized by reduced vocal intensity, monotony, hoarseness, breathiness, vocal tremor, and imprecise articulation. These alterations result from deficits in the amplitude, speed, stability, precision, and coordination of speech-related movements, largely due to rigidity and bradykinesia affecting the respiratory, laryngeal, and articulatory musculature. Consequently, patients often present with reduced respiratory support, impaired vocal fold adduction, and altered voice resonance. Additionally, reduced self-perception of vocal intensity is frequently observed, further compromising effective communication. 10 11 12 13

### Living with dysarthria (LwD) questionnaire

The LwD questionnaire was developed and validated by Hartelius et al. 14 as a patient-reported outcome measure designed to assess the impact of dysarthria on daily life from the patient's perspective. The instrument was created to capture not only speech motor impairments but also their social, emotional, and functional consequences. Its original validation included 55 individuals with varying degrees of dysarthria and demonstrated that perceived communication difficulties were not necessarily correlated with clinical severity. Reported challenges were primarily related to restrictions in social participation, particularly in professional and educational settings, as well as difficulties in expressing one's personality. 14

The questionnaire has also been translated and culturally adapted to different contexts, including a recent validation to Portuguese by Holanda et al., 15 supporting its applicability in broader clinical settings.

Preliminary data presented in a conference abstract by Puhl et al. 16 suggested that the LwD questionnaire may be useful in identifying communication difficulties in individuals with IPD from the patient's perspective, reinforcing the importance of assessments that incorporate both motor and psychosocial dimensions of dysarthria.

The LwD consists of 50 items distributed across 10 domains addressing various aspects of speech production, communication, and the influence of social, behavioral, and environmental factors. Responses are rated on a 6-point Likert scale ranging from “totally disagree” (1) to “totally agree” (6), with total scores obtained by summing individual item responses (50–300).

Based on this structure, the questionnaire assesses multiple domains affected by dysarthria, including communication, social relationships, self-esteem, emotional aspects, and participation in daily activities. Its main advantage over other speech assessment tools lies in its patient-centered approach, capturing the subjective experience and psychosocial impact of dysarthria in everyday life. Unlike traditional clinical assessments that predominantly focus on motor aspects, the LwD enables a broader understanding of the challenges faced by individuals, facilitating more targeted therapeutic interventions.

## METHODS

This study is a narrative review aimed at summarizing the available evidence on the use of the LwD questionnaire in individuals with IPD.

A literature search was conducted in the SciELO and PubMed databases, including studies published between 2015 and 2024. The search strategy combined the following terms: ( *Idiopathic Parkinson's disease* OR *parkinsonism* ) AND ( *dysarthria* ”) AND ( *Living with Dysarthria* ) AND ( *Quality of Life* ). Additionally, one study 17 presented at the XXXI Brazilian Congress of Neurology (2024) was included.

Studies were considered eligible if they included patients with IPD; applied the LwD questionnaire; and evaluated outcomes related to QoL or communication.

The exclusion criteria were studies not involving dysarthria or not using the LwD instrument.

The screening process was conducted in two stages: initial title and abstract screening followed by full-text review. A total of five studies met the criteria for inclusion in this review ( Table 1 ).

**Table 1:** Summary of studies on LwD in dysarthria included in the review

Study (year)	Population	Tool	Main findings	Severity/QoL
Costa et al. (2016) 4	33 with IPD + 40 controls	LwD	Dysarthria associated with worsening QoL, emotional domain most affected	Greater impact on cases with more vocal symptoms
Lirani-Silva et al. (2015) 3	13 with IPD + 12 controls	Acoustic analysis + LwD	Changes in prosody and vocal frequency had a greater impact on QoL	Correlation with early stages and non-motor symptoms
Diaféria et al. (2017) 2	16 with IPD	LwD + Self-assessment	Group therapy with vocal coaching improved communication and reduced social avoidance	Improvements observed regardless of motor severity
Rohers et al. (2022) 18	1 with IPD 1 with ALS	LwD + FFR	Demonstrated correlation between LwD and electrophysiological speech measures	Suggested association between dysarthria and neurophysiological decline
Moraes et al. (2024) 17	25 with IPD	LwD, UPDRS, Hoehn & Yahr	UPDRS showed a significant correlation with LwD	Significant correlation between dysarthria severity and decreased QoL

Abbreviations: FFR, frequency-following response; IPD, idiopathic Parkinson's disease; LwD, living with dysarthria; QoL, quality of life; UPDRS, unified Parkinson's disease rating scale.

Data extraction included study characteristics, such as sample size, patient demographics, clinical features, and main outcomes related to dysarthria and QoL. A qualitative assessment of methodological aspects, including sample size, study design, and potential sources of bias, was performed. Although the primary focus of this review is IPD, one study involved one patient diagnosed with amyotrophic lateral sclerosis (ALS), which may introduce heterogeneity and limit disease-specific conclusions.

### Population

The included studies comprised patients diagnosed with mild to moderate dysarthria associated with IPD. Sample sizes ranged from 2 to 33 participants. Overall, the studies included approximately 141 individuals, comprising 52 healthy controls, 88 patients with IPD, and 1 patient with ALS. Participants' ages ranged from 29 to 99 years. When available, studies included both male and female participants and were conducted in different geographical settings, although detailed demographic distribution was not consistently reported across all studies.

### Synthesis of the reviewed studies

The analyzed studies provide a broad overview of how dysarthria affects different dimensions of patients' lives. All studies demonstrated that worsening vocal symptoms in IPD is strongly associated with a decline in QoL, particularly in the emotional domain. When comparing IPD patients to healthy adults, the studies showed that changes in prosody and vocal frequency control emerge early and are directly linked to reduced QoL.

Diaféria et al. 2 introduced a practical perspective by demonstrating that group therapy programs based on vocal coaching improve communication and help reduce social isolation.

Moraes et al. 17 analyzed 25 IPD patients using the LwD and the correlation between its scores and the Unified Parkinson's Disease Rating Scale (UPDRS) and Hoehn & Yahr stages. Findings showed that patients with dysarthria in IPD exhibited slower speech, frequent need for repetition, impaired self-esteem, a tendency to avoid social interactions, and that there is a direct correlation between symptom severity, mainly measured by the UPDRS, and worse QoL as identified by the LwD. 17

Finally, Rohers et al. 18 offered a complementary perspective by correlating LwD results with electrophysiological measures of speech, further strengthening the credibility of the questionnaire as an instrument that reflects not only the patient's perception but also objective changes in the speech motor system. 18

## DISCUSSION

### Main findings

The findings of this review suggest that dysarthria in patients with IPD extends beyond the motor system, profoundly affecting self-esteem, social participation, and emotional well-being. The relationship between the severity of speech impairment and the decline in QoL appears consistently across studies, with evidence suggesting that emotional impacts may arise even in the early stages of the disease.

Changes in prosody and vocal frequency control were identified as significant signs of communicative impairment. At the same time, findings from intervention studies reinforce the need for broader therapeutic approaches that go beyond motor aspects, incorporating strategies to strengthen functional communication and reduce social isolation. Furthermore, findings correlating patient-reported outcomes with objective speech measures reinforce the clinical relevance of tools such as the LwD questionnaire in capturing both subjective and measurable aspects of communication impairment.

These findings are consistent with previous literature emphasizing the multidimensional impact of dysarthria in IPD and the importance of incorporating patient-reported outcomes into clinical assessment, particularly to capture psychosocial burden and communicative participation. 19 20

### Clinical relevance of the LwD questionnaire

Dysarthria is a frequent and often disabling manifestation of IPD, affecting up to 70 to 90% of patients and significantly compromising verbal communication. 2 3 4 Hypokinetic dysarthria, the most common subtype in IPD, is characterized by reduced vocal intensity, monotony, and imprecise articulation, which directly impact speech intelligibility and communicative effectiveness. 21

These impairments extend beyond speech production, influencing emotional expression, social interaction, and overall autonomy. As communication becomes less effective, patients may experience frustration, embarrassment, reduced self-esteem, and social withdrawal, which can contribute to anxiety and depressive symptoms. 5

In this context, assessing the impact of dysarthria on QoL is essential for guiding clinical management. Traditional clinician-based evaluations, which primarily focus on motor aspects of speech, may fail to capture the patient's lived experience. The LwD questionnaire addresses this gap by providing a patient-reported assessment of the psychosocial and functional consequences of dysarthria.

The LwD is also easy and quick to apply, which makes it very practical in clinical settings. It allows health professionals, especially speech therapists and physicians, to understand the patient's difficulties more clearly and decide when to refer them for more detailed evaluations or early interventions. Speech therapy, when started in time, can not only improve speech but also help restore confidence, reduce isolation, and enhance QoL. Evidence from recent studies highlights that interventions such as speech-language therapy, including approaches like the Lee Silverman Voice Treatment (LSVT), may improve vocal intensity, intelligibility, and communicative participation in individuals with IPD. 22 23 24

### Integration with the clinical context

Beyond communication impairment, dysarthria in IPD often coexists with other clinically relevant conditions, such as dysphagia. Dysphagia represents a significant risk factor for complications including malnutrition, dehydration, and aspiration pneumonia, which remains one of the leading causes of hospitalization in this population. Early identification of speech and swallowing impairments is therefore essential for comprehensive patient management.

These findings reinforce the importance of integrated clinical approaches that consider both motor and non-motor aspects of the disease, highlighting the role of multidisciplinary care in improving patient outcomes. Recent large-scale clinical trials have also emphasized the relevance of structured speech therapy interventions in improving patient-centered outcomes, further supporting the integration of communication-focused strategies into routine care. 25 26

### Study limitations

Despite its relevance, this topic is still poorly explored. Only five studies were found that evaluated dysarthria in IPD and its impact on QoL using the LwD questionnaire, which shows how little this subject has been investigated so far. These studies are characterized by small sample sizes, methodological heterogeneity, and variability in assessment approaches, which limit comparability and generalizability. Additionally, most studies do not explore the relationship between speech impairment and broader clinical outcomes, such as swallowing dysfunction, hospitalization rates, or long-term QoL measures, further restricting the interpretation of findings. This lack of standardization limits comparability across studies and reduces the robustness of the synthesized findings.

Second, the overall body of evidence remains limited, with a small number of studies specifically investigating the application of the LwD questionnaire in individuals with IPD and dysarthria. Furthermore, some of the available evidence is derived from preliminary data, including conference abstracts, which may lack detailed methodological reporting and peer-reviewed validation.

Third, the review was not conducted as a systematic review, and no formal meta-analysis was performed, which may introduce selection bias and limit reproducibility. Furthermore, the inclusion of studies involving heterogeneous neurodegenerative conditions may restrict the ability to draw disease-specific conclusions for IPD. These limitations may affect the internal and external validity of the findings, reinforcing the need for cautious interpretation.

## Future directions

Given this scenario, there is a clear need for more research in this area. Future studies should include larger and more diverse samples, adopt standardized methodologies, and investigate longitudinal outcomes. It is also important for future research to focus on patients' perspectives and on how speech difficulties affect daily life. This can help improve the care provided and ensure that it meets the real needs of patients with IPD.

Despite the limited evidence base, this review contributes by synthesizing current knowledge on the use of a patient-reported outcome measure specifically focused on dysarthria, highlighting gaps and guiding future research directions.

The application of LwD in patients with IPD may represent a useful patient-centered approach. More than merely measuring motor speech impairments, based on perception of their difficulty, the questionnaire captures patients' subjective experience by investigating how these limitations affect their social life, emotional well-being, and interpersonal relationships.

Moreover, its brief and practical application facilitates incorporation into clinical routines, especially in neurology, supporting clinical reasoning by providing valuable information to categorize speech symptoms, assess perceived severity, and guide referrals to speech therapy, otolaryngology, or psychology.

It is also important to emphasize the value of acknowledging patients' perspectives on this impactful condition, which can be accessed through a clear, objective instrument with accessible language and straightforward questions.

Finally, considering that dysarthria compromises not only speech intelligibility but also the execution of social functions and psychological well-being, the use of the LwD contributes to a more patient-centered care model, addressing their real needs and promoting a broader improvement in QoL.
